# In Vitro Anti-Aging Properties of a *Cetraria islandica* Extract: Interest for Skin Care Cosmetics

**DOI:** 10.3390/molecules31172988

**Published:** 2026-08-26

**Authors:** Anthony Groso, Paul Jabet, Yang Fan, Wang Hua, Hu Yawen, Guo Miao, Richard Daniellou, Guillaume Collet

**Affiliations:** 1 Cosmetology Department, AgroParisTech, 45100 Orléans, France; 2 Research & Development Center, Mageline Biology Tech Co., Ltd., Wuhan 430060, China; 3 National Research Institute for Agriculture, Food and the Environment (INRAE), AgroParisTech, UMR Micalis, Universite Paris-Saclay, 78350 Jouy-en-Josas, France

**Keywords:** *Cetraria islandica* hydroglycolic extract, skin aging, antioxidant, anti-inflammatory, dermal matrix remodeling, tyrosinase

## Abstract

Skin aging is a major consideration regarding cosmetic skin care. The cosmetic field is seeking new active ingredients able to slow down the appearance of signs of age. Some lichens and more specifically the genus *Cetraria s. str*. presents an interest as a source of active molecules because it is already being used in traditional and modern medicines. To better evaluate the potential of *C. islandica* as anti-aging skin care, we investigated some key aspects to protect skin from external aggression and to avoid skin alteration, which would exacerbate signs of age. Among them, our results reveal (i) antioxidant capacities; (ii) anti-inflammatory properties on human keratinocytes once stimulated, as shown by a decrease in IL-6, IL-8, CXCL9, CXCL10, and CCL5; (iii) a downregulation of MT1-MMP, which corresponds to decreased tissue remodeling; and (iv) an inhibition of human tyrosinase, which could correct the appearance over time of age-related skin spots. Altogether, these results highlight the potential use of *C. islandica* in anti-aging skin care.

## 1. Introduction

Aging is a natural phenomenon occurring over time for all living species. For human standards of beauty, it remains unwanted and can lead to social discrimination. This is the reason why the cosmetic field aims at delaying its appearance, correcting it when already present, or even masking it [1]. For this purpose, an extensive part of research is devoted to finding new active compounds and to developing new products. Signs of ageing appear generally around 20 years old and are the consequence of molecular and cellular changes occurring within the skin. Oxidative stress is found among some of the aggravating factors, explaining why a lot of cosmetic skin care contains antioxidants [2]. Similarly, inflammation is also deleterious to skin health and quality. The skin is constantly exposed to aggression such as cold temperature, sun exposure, and/or pollution [3]. These aggressions create cell and molecular lesions, which mobilize the inflammation to induce reparation. Unfortunately, the resulting skin remodeling exacerbates signs of aging, justifying why skin inflammation is wished to be low to keep it youthful [4,5].

The constant quest for new active ingredients, lichens, and more specifically the genus *Cetraria s. str*. (*Cetraria islandica* (L.) Acharius s.l. (known as Iceland moss), *Parmeliaceae* family, Cetrarioid clade) has been widely employed in traditional and modern medicines and has an important nutritional value in numerous countries [6,7]. Among the 15 species that compose the genus *Cetraria*, the lichen *Cetraria islandica* has been described for its numerous biological properties such as immunological, immunomodulating, antioxidant, antimicrobial, cytotoxic, genotoxic and antigenotoxic, and anti-inflammatory activities. These properties justify the interest from many industries for selling it as medicines, dietary supplements, daily herbal drinks, and cosmetics [6,8]. Indeed, as an active ingredient in cosmetic skin care products, *C. islandica* extracts exist on the market in various forms, including aqueous, glycerin, or propylene glycol solutions (CAS 84776-25-0, INCI Name: *Cetraria Islandica* Extract). Among reported functions, it is mainly used for cleansing, skin conditioning—emollient, and smoothing and soothing as listed in the COSMILE Europe or in the CosIng—Cosmetics Ingredients Database.

In the herein reported publication, *C. islandica*, and more specifically a hydroglycolic extract of *C. islandica*, is evaluated as a potential candidate for anti-aging cosmetic skin care with a deep focus on antioxidant and anti-inflammatory properties. To the best of our knowledge, the results obtained at the skin cell level remain sparse across the literature. Consequently, these experiments are conducted on keratinocytes, which correspond to the first cell type exposed to external aggression and consequently the cells that a cosmetic care will try to protect. The present study aims to bridge the gap between in vivo effects of *C. islandica* extract-containing cosmetics products and reported molecular antioxidant properties. Obtained results will reinforce both traditional and modern uses by cellular evidence on keratinocytes while shedding light on cell-mediated molecular mechanisms. Ultimately, the results will constitute a solid base for further use of *C. islandica* in cosmetics as active ingredient.

More precisely, this work first aims to evaluate at both the molecular and cellular level the ability of the *C. islandica* extract to prevent an oxidative stress known to be deleterious for skin health. A second aspect of the work quantifies how efficient the *C. islandica* extract is to decrease an inflammatory response upon a pro-inflammatory stimulation. For this purpose, five cytokines and four pro-inflammatory chemokines, already reported in inflammatory skin diseases, were quantified to obtain a comprehensive answer about the response to an inflammatory stress [9]. Aside from these two aspects, the quality of the skin matrix is evaluated with the relative quantification of MT1-MMP (Membrane Type 1 Metalloproteinase) expression by flow cytometry. Lowering the quantity of enzymes like MTI-MMP, which are able to degrade skin elements such as collagen, would directly contribute to maintaining skin quality and health. In skin epidermis, MT1-MMP has been shown to be involved in tissue remodeling events ranging from tumor invasion and angiogenesis to growth and development [10]. Nevertheless, most published works focus on lesional skin, including melanocytic nevi [11] and tumor [12,13,14] with a tight link with cell migration and survival [10]. Skin pigmentation was also considered in this study, aware of the skin dysregulation of melanin production over age with appearance of hyper-pigmentation skin spots [15,16]. Consequently, an evaluation of the inhibitory properties of *C. islandica* extract is performed here on human tyrosinase enzyme, the key enzyme involved in melanin synthesis.

## 2. Results and Discussion

**Figure 1:**
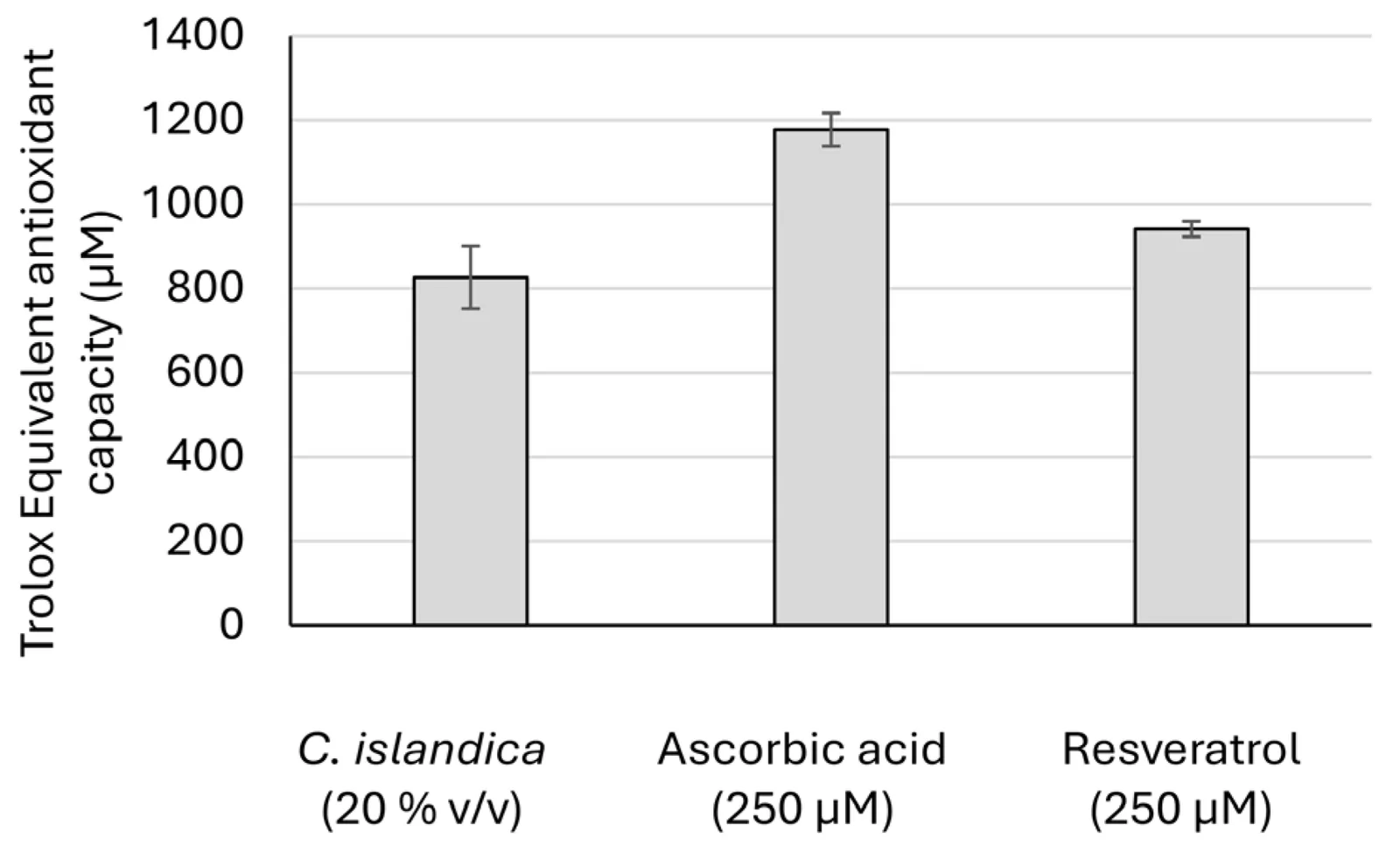
Antioxidant activities of *C. islandica* hydroglycolic extract (20% *v*/*v*) compared to resveratrol (250 µM) and ascorbic acid (250 µM). Antioxidant capacity (µM). Mean ± SD (*n* = 3).

The antioxidant activity of the new *C. islandica* hydroglycolic extract was measured in vitro to assess its efficacy in preventing oxidative stress, the main process that in vivo is well known to significantly affect the health of the skin. The measure uses a copper-based method similar to CuPRAC assay [17]. The method used in this study evaluates the transition metals reducing power and free radical capturing effect of the plant extract. Antioxidant results presented in Figure 1 were determined as Trolox equivalents and are expressed in TEAC for Trolox Equivalent Antioxidant Capacity (in µM). The TEAC determined for our *C. islandica* hydroglycolic extract is around 800 µM of TEAC at 20% (*v*/*v*). Ascorbic acid displays a 1200 µM of TEAC at 250 µM, whereas resveratrol presents a 950 µM of TEAC at the same molar concentration (Figure 1). While special attention should be paid to the concentration units, these results indicate a high antioxidant activity of the *C. islandica* hydroglycolic extract considering already known and published ascorbic acid and resveratrol [18,19]. Its efficacy is comparable to these two well-known antioxidant molecules used as references (Figure 1). While different in terms of preparation and concentration, our results are completely in adequation with the literature and confirm the antioxidant activity of *C. islandica* [20,21].

Further studies were conducted on cell cultures using living keratinocytes and, more precisely, the human HaCaT keratinocyte cell line. The choice of this cell type is justified because keratinocytes are the first cells of the skin exposed to external aggression, including, for example, UV, temperature, or pollution [3]. These aggressions lead to oxidative stress and inflammation, which directly impact skin health and contribute to accelerating signs of aging [22,23]. This is particularly true considering the life span of a person and all the repeated aggressions to which the skin is exposed.

**Figure 2:**
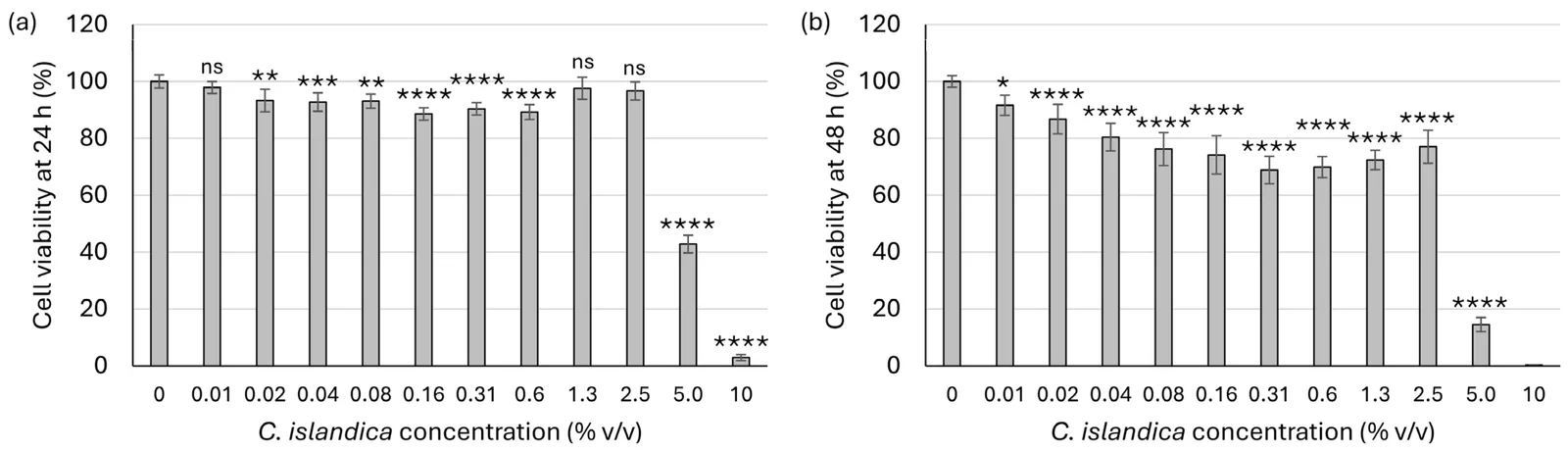
The cell cytotoxicity experiment performed on HaCaT cells. *C. islandica* hydroglycolic extract, diluted in complete cell culture medium, was tested at different concentration and was incubated for (**a**) 24 h and (**b**) 48 h with cells. Data are normalized on untreated cells considered as 100% viability. Error bars represent the standard deviation (*n* = 8). The degree of significance was obtained by one-way ANOVA analysis. * *p* < 0.05, ** *p* < 0.01, *** *p* < 0.001, and **** *p* < 0.0001 were considered to indicate statistically significant difference compared to the 100% viability set without the addition of extract (0% *v*/*v*). “ns” for no statistical significance correspond to *p* > 0.05.

Prior cell experiments on the HaCaT keratinocyte cells line, cytotoxicity experiments were carried out to determine the dose limit of use (DLU) for *C. islandica* hydroglycolic extract. These experiments aim to set the maximum concentration to be applied on living cells without inducing cell death. Considering cumulative results obtained at 24 and 48 h of incubation with the extract, the DLU is determined to be at 2.5% (*v*/*v*) for a threshold fixed at 90% viability (Figure 2).

Once determined, the DLU was used to evaluate if *C. islandica* hydroglycolic extract could be useful to promote skin health and to reduce signs of aging by decreasing the inflammatory response of skin upon aggression. This anti-inflammatory property was assessed through the ability of *C. islandica* hydroglycolic extract to decrease the amplitude of a cell response to a pro-inflammatory stimulus achieved by IFNγ and TNFα. The cell response was followed by the quantification of pro-inflammatory messengers, including cytokines and chemokines, respectively, IL-6, IL-8, CXCL9, CXCL10, and CCL5 [9]. ELISA was the selected method to quantify these pro-inflammatory proteins in the supernatant.

The stimulation was validated by the observation of a clear overexpression of all 5 pro-inflammatory messengers upon IFNγ and TNFα stimulation. Experiments were performed at six different concentrations, including DLU, DLU/2, DLU/4, DLU/8, DLU/16, and DLU/32. While performed, ELISA assay did not reveal the presence of IL-33 independently to the stimulation. This absence of detection indicates either that the human HaCaT keratinocyte cell line does not secrete a detectable amount of IL33 or that a stimulation with IFNγ and TNFα does not induce its secretion in a detectable amount. IL-1β, CCL17, and IL-18 were detected with an effective stimulation with IFNγ and TNFα. However, quantifications were too close to limits of detection, leading to unreliable results, which were consequently not presented.

As a control, the addition of *C. islandica* hydroglycolic extract alone does not induce a secretion of the studied pro-inflammatory messengers (Supplementary Figures S1–S5).

**Figure 3:**
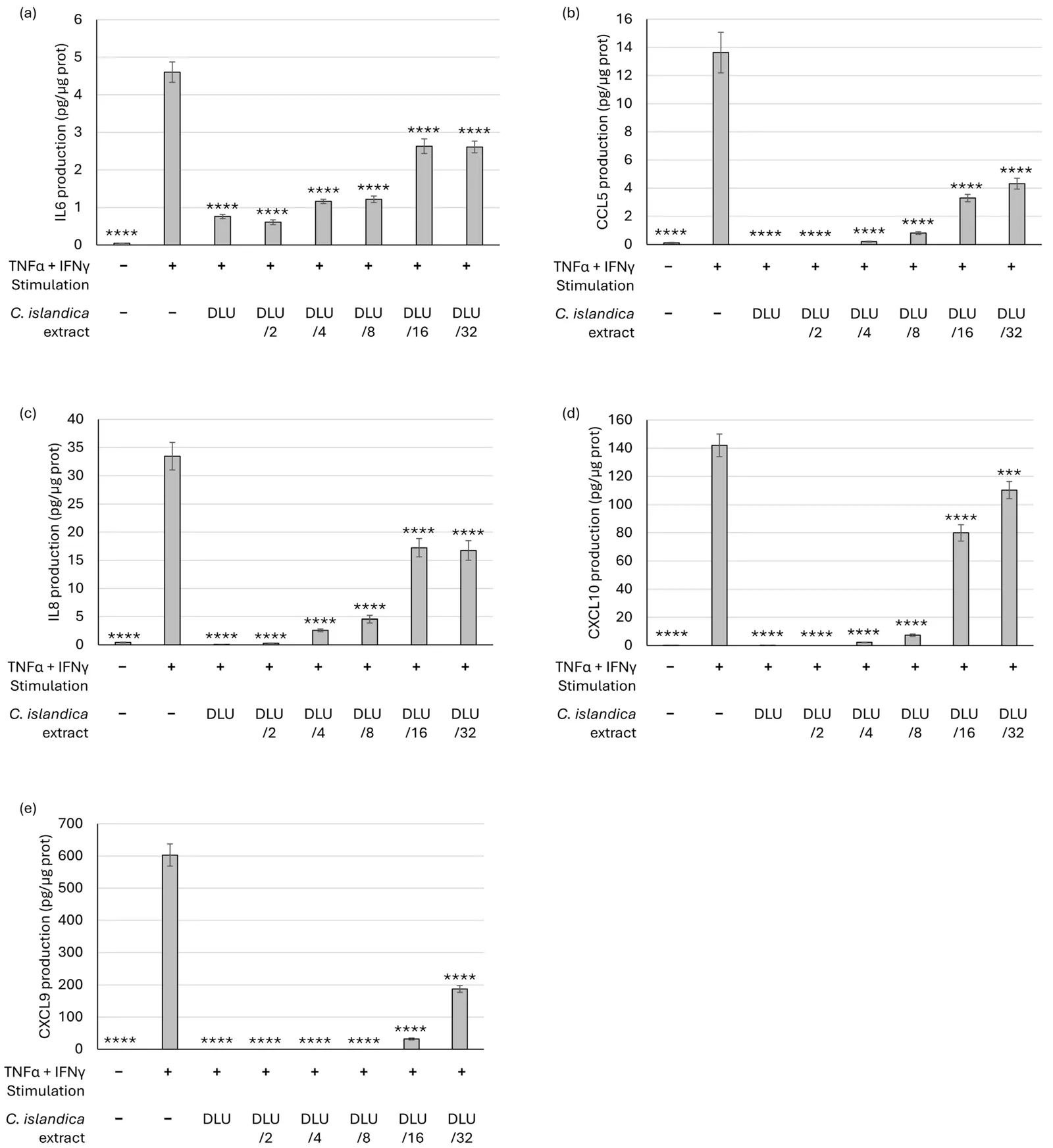
The quantification of pro-inflammatory cytokine/chemokine production by HaCaT cells, challenged or not with TNFα + IFNγ (10 ng/mL) and incubated with various concentrations of *C. islandica* hydroglycolic extract for 48 h. The production of (**a**) IL6, (**b**) CCL5, (**c**) IL8, (**d**) CXCL10, and (**e**) CXCL9 was measured by ELISA. All results are normalized by cellular proteins amount. Data are expressed in ng of cytokines/chemokines per mg of cell proteins. Values are means ± SD (N = 3, *n* = 1). The degree of significance was obtained by one-way ANOVA analysis. *** *p* < 0.001 and **** *p* < 0.0001 were considered to indicate statistically significant differences compared to the stimulation of only condition.

Noteworthily, a clear decrease for all five pro-inflammatory messengers was observed at the DLU as compared to the stimulated condition only (Figure 3). Interestingly, a complete inhibition was observed for IL-8 (Figure 3c), CXCL9 (Figure 3e), CXCL10 (Figure 3d), and CCL5 (Figure 3b), highlighting the strong efficacy of *C. islandica* hydroglycolic extract. In addition, an interesting dose–response effect was observed with all quantifications allowing to unambiguously link the observed anti-inflammatory effect with the presence and amount of *C. islandica* hydroglycolic extract (Figure 3). Even diluted, the extract shows an impressive inhibition of the pro-inflammatory protein productions up to a factor 32 dilution, corresponding to a concentration of 0.078% (*v*/*v*). The effect is particularly visible for CCL5 (Figure 3b) and CXCL9 (Figure 3e) highlighting again how efficient *C. islandica* hydroglycolic extract in a dose of 2.5 a,d 1.25% (*v*/*v*) is in decreasing the cellular inflammatory response. To the best of our knowledge, this anti-inflammatory aspect of *C. islandica* was never investigated so finely, with the quantification of five pro-inflammatory mediators. Only traditional methods used in medicine were documented with anti-inflammatory applications [6,24].

**Figure 4:**
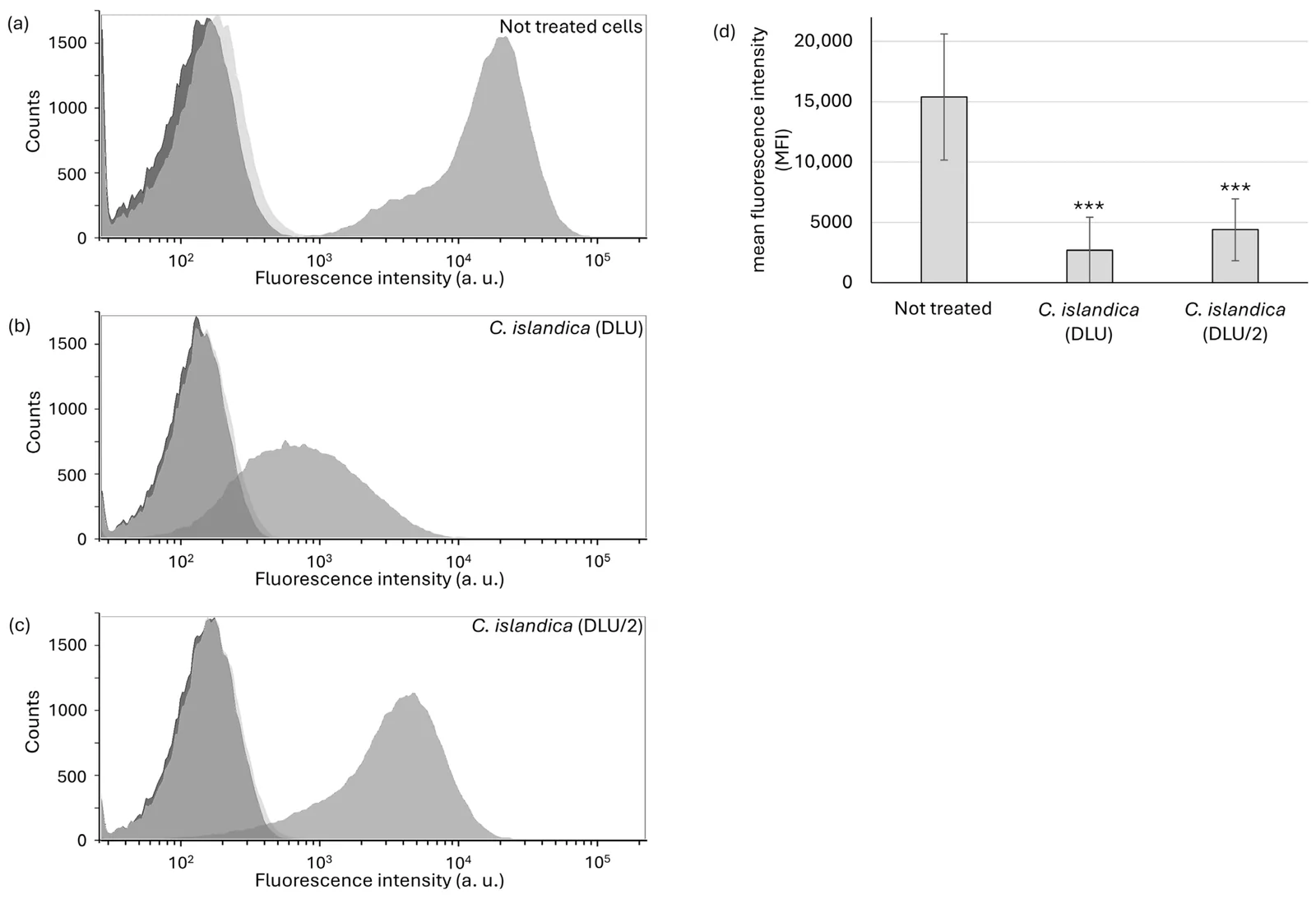
A representative histogram of the flow cytometry analysis (FACS) of the HaCaT cells for MT1-MMP protein expression of untreated cells (**a**) or 48 h incubation with *C. islandica* hydroglycolic extract at the DLU (**b**) or DLU/2 (**c**). White cells are presented in black, isotypic control in clear gray, and MT1-labeled cells in gray. (*n* = 10,000). (**d**) The quantification of MT1-MMP protein expression by HaCaT cells assessed by flow cytometry after 48 h of incubation with *C. islandica* hydroglycolic extract. The results are expressed as expression fold change relative to untreated cells. The extract was investigated at 2.5% (*v*/*v*) for the DLU and 1.25% (*v*/*v*) for the DLU/2. (N = 5, *n* = 10,000). The degree of significance was obtained by one-way ANOVA. *** *p* < 0.001 were considered to indicate statistically significant difference compared to untreated cells.

Considering the importance of skin extracellular matrix (ECM) in the aging process, another in vitro investigation was carried-out on membrane-type I matrix metalloproteinase, also called MT1-MMP/MMP14, which in vivo plays an important role in ECM remodeling by being able to degrade type-I collagen, activate pro-MMP-2, and process cell adhesion molecules such as CD44, E-cadherin, and integrin alpha V [25]. Moreover, MT1-MMP has been shown to be a crucial enzyme that maintains fibrillar collagen homeostasis [26]. The alterations made to the ECM by MMPs might contribute to skin wrinkling, a characteristic of premature skin aging [25]. Never reported so far, the modulation of MT1-MMP protein expression on HaCaT cells’ surface was assessed by flow cytometry after 48 h of incubation with *C. islandica* hydroglycolic extract. The results (Figure 4) are expressed as mean fluorescence intensity regarding not treated cells (Figure 4a). In each case, the *C. islandica* hydroglycolic extract was investigated at 2.5% (*v*/*v*) corresponding to DLU (Figure 4b) and 1.25% (*v*/*v*) corresponding to DLU/2 (Figure 4c). The results presented in Figure 4 (and Figure S6) show a strong downregulation of MT1-MMP on the surface of HaCaT when incubated with *C. islandica* hydroglycolic extract with a 6-fold decrease at the DLU and 3.5-fold at DLU/2, respectively. This change, once the extract is diluted, highlights the dose–response effect of the extract. While commonly associated with dermis and its firmness, the present study focuses on epidermis with its keratinocytes, where signs of age take another form. Indeed, within this skin layer and over time, the same and well-known disequilibrium will occur with (i) an increasing action of matrix-degradation enzymes together with (ii) a decrease in synthesis of new matrix components. The increase in MT1-MMP activity with aging has been clearly shown in some tissues in physiological and pathological contexts [25,27,28,29,30]. Within the epidermis, aging-induced disequilibrium will disturb skin homeostasis, skin repair, and keratinocytes migration/renewal [31,32]. Moreover, MT1-MMP has been shown to activate secreted MMP-1 and MMP-2, which will also degrade other ECM components [25]. Nevertheless, MT1-MMP also plays important physiological roles in tissue remodeling, epidermal homeostasis, and wound repair. Therefore, the decreased expression of MT1-MMP on the HaCaT cell surface when incubated with *C. islandica* hydroglycolic extract must be considered carefully with particular attention paid to biological complexity.

Among other signs appearing over aging, some people develop pigmentation disorders with the appearance of spots due to a lack (white spot) or an accumulation of melanin (dark spot). Generally, hypo- or hyperpigmentation spots do not affect the entire body, and they are under genetic control, involving ethnicity [15,16]. When affecting face and hands, this skin dysregulation is finding corrective measures by the modulation/regulation of the tyrosinase, which is the limiting enzyme in the synthesis pathway of melanin [33].

**Figure 5:**
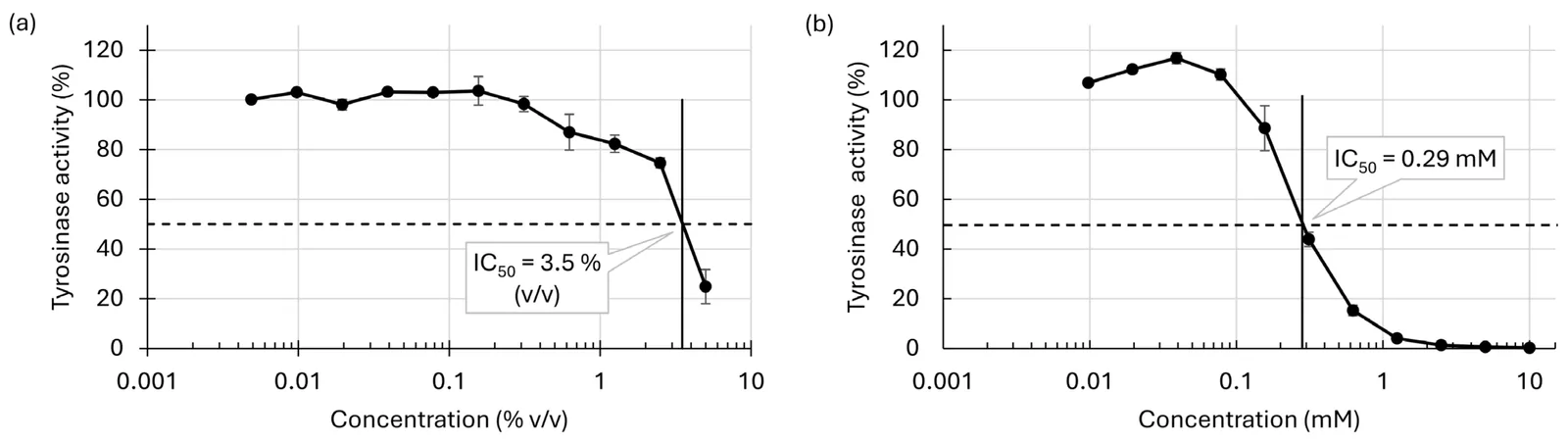
The inhibition of tyrosinase activity by (**a**) *C. islandica* hydroglycolic extract and (**b**) kojic acid in cell lysate derived from the human MNT-1 melanoma cell line. Cell lysates were incubated with various concentrations (% *v*/*v*) of either *C. Islandica* hydroglycolic extract or kojic acid solution. Conditions without extract/molecule (vehicle only) were used as the control to define 100% activity. The dashed lines represent 50% of tyrosinase activity. Data are shown as means ± SD. (*n* = 3).

The potential activity of *C. islandica* to modulate the tyrosinase activity was evaluated by an in vitro inhibition assay using a human tyrosinase. The human melanocyte cell line MNT-1 is the cell type used as a source of tyrosinase in this assay [34]. Figure 5a shows an inhibition of tyrosinase at a high concentration of *C. islandica* hydroglycolic extract. Kojic acid presented in Figure 5b was used as reference for tyrosinase inhibition and as positive control for the assay. An IC_50_ (half-maximal inhibitory concentration) of 3.5% (*v*/*v*) was determined, which means that the tyrosinase activity is decreased to 50% at this concentration. This result confirms the tyrosinase inhibition observed by Malaspina et al. and brings one additional demonstration [6,35]. This result clearly indicates a promising aspect of *C. islandica* in skin aging with a valorization in pigmentation disorders and age-related hyper-pigmentation spot appearance. While this aspect is addressing melanocytes, the range of concentration showing an effect on the enzyme is close to the range presenting a cell toxicity on human HaCaT keratinocyte. Consequently, particular attention will have to be paid to cell toxicity considering further application in vivo and regarding the concentration of extract in the final formulated product.

New products are continuously released on the market for the enhancement of both skin health and skin beauty. Specific attention is paid to mature skin and aging, which involve functional slowdown combined with environmentally induced alterations. In the quest for new actives, a focus was made herein on *C. islandica*, investigating how useful an extract of this specific lichen could be on skin and more specifically on skin aging. *C. islandica* demonstrates three different promising properties acting simultaneously as (i) antioxidants and (ii) anti-inflammatory substances to protect skin from aggressions and (iii) as a modulator of skin pigmentation to correct age-related skin spots.

Whereas almost no studies were carried out in vitro on living skin cells, the presented results shed light on cell-based molecular mechanisms. The antioxidant effect on HaCaT keratinocytes reveals ROS-scavenging effects upon H_2_O_2_-induced oxidative stress. The anti-inflammatory effect has been shown to be extremely efficient regarding the production of five mediators of inflammation produced by keratinocytes once stressed. The potential to attenuate age-related skin spots was shown by an inhibitory effect directly on the key enzyme responsible for the synthesis of melanin pigments.

Interestingly, experiments were carried out with a whole natural extract of *C. islandica* (non-standardized for a single active molecule) to preserve all the extract complexity. Nevertheless, it could constitute a possible limitation in a way where the direct link between a molecule and its activity is not provided.

Regarding modern cosmetic preoccupations and social pressure to keep skin young as long as possible, the present study brings new evidence in the field of cosmetics to those who would like to valorize the potential of *C. islandica* hydroglycolic extract as a potent ingredient to alleviate signs of skin aging.

Nevertheless, skin aging is a complex phenomenon deeply affecting skin. Other markers of skin aging could be studied to assess the extent to which C. islandica is effective in reducing the signs of aging. Moreover, further studies are required, especially considering that reported investigations were carried out in vitro. The use of more elaborated models such as reconstructed human skin models, ex vivo skin models, skin penetration assays, or even clinical studies would be useful to strengthen cosmetic evidence of efficacy.

## 3. Materials and Methods

Materials. The ingredient used was a commercially available *C. islandica* extract supplied by GreenTech Biotechnologie (iceland moss concentrated hydroglycolic extract, ref 3001293, Biopôle Clermont-Limagne, 2 Rue Marie Curie, 63360 Saint-Beauzire, France, https://www.greentech.fr/en/, accessed on 1 August 2026), utilized according to its standard Product Information File (PIF) specifications. *C. islandica* lichens used by GreenTech come from Europe (Romania, Ukraine, Bulgaria). The ingredient “*C. islandica* extract” is composed of propylene glycol (CAS-No.: 57-55-6) 40–60%, water (CAS-No.: 7732-18-5) 40–60%, and *C. islandica* extract (CAS-No.: 84776-25-0, INCI Name: *Cetraria Islandica* Extract) 1–10%. The typical physicochemical characteristics of the ingredient are physical state: liquid; color: light amber to dark brown; relative vapor density at 20 °C: 1.01–1.06; solubility: water soluble; and pH: ranges between 4 and 6 to match standard cosmetic compatibility. The extract was used as received and stored at room temperature. An HPLC chromatogram is provided in Figure S7 together with its experimental details in Figure S8.

Antioxidant assay. Antioxidant properties of *C. islandica* extract were determined using the Antioxidant Assay Kit provided by Merck (Darmstad, Germany, reference MAK334-1KT) and realized according to manufacturer instructions. Briefly, 20 µL of sample and standard (Trolox, range from 1.5–1000 µM) were dispensed in a flat bottom 96 well plate. Then, 100 µL of reaction mix was added to all assay wells. After a short incubation of 10 min at room temperature, absorbance was read at 570 nm (A_570_) with a Tecan INFINITE M PLEX 200 Pro plate reader (Tecan, Mannedorf, Switzerland). The Trolox Equivalent Antioxidant Capacity (TEAC) of *C. islandica* extract was calculated as follows:

$$TEAC\left(µM\right)=\frac{{\left(A_{570}\right)}_{sample}-{\left(A_{570}\right)}_{blank}}{Standard\;Trolox\;slope\;({µM}^{-1})}\;\times n$$

where (A_570_)_sample_ = the absorbance of the sample, (A_570_)_blank_ = the absorbance of the medium blank, and n = sample dilution factor. Standard Trolox slope was determined from the plot of the ∆A_570_ ((A_570_)_sample_ − (A_570_)_blank_) against Trolox concentrations. Ascorbic acid (Sigma-Aldrich, Saint-Louis, MO, USA, A2218) and resveratrol (Sigma-Aldrich, R5010) were added to the assay as reference molecules with antioxidant properties.

Cell culture. HaCaT cells, a keratinocyte cell line, was provided from CLS company (CLS [Cell Line Service], Cytion GmbH, Heidelberg, Germany, batch number 300493-4820). MNT-1 cells, a human melanoma cell line was provided FROM ATCC company (ATCC, Manassas, VA, USA, ATCC-CRL-3450, batch number 70050224). The two cell lines were cultured in Dulbecco’s Modified Eagle’s Medium (DEMEM, Gibco) supplemented with 10% (*v*/*v*) Fetal Bovine Serum (FBS, Gibco, Grand Island, NY, USA), 100 U/mL penicillin (Sigma), and 100 μg/mL streptomycin (Sigma). Cells were routinely cultured at 37 °C in a humidity-saturated incubator in a 95% air/5% CO_2_ atmosphere and passaged by detaching cells with trypsin-EDTA solution (Gibco) either at 0.05% for MNT-1 cells or at 0.25% for HaCaT cells.

Human tyrosinase inhibition assay. The method was adapted from Roulier et al. [34]. Briefly, an amount of 10 × 10^6^ MNT-1 cells was collected by detaching them with 0.05% trypsin-EDTA (*w*/*v*). The cells suspension was washed 2 times by centrifugation (300 *g*, 5 min) with phosphate buffer saline (PBS 1X). After a final centrifugation at 300 *g* for 5 min, the supernatant was discarded and the cell pellet was dispersed in 2 mL lysis buffer (PBS with 1% (*v*/*v*) Triton X-100). The lysis was achieved with homogenization by pipetting for 5 min at 4 °C. After the lysis, the volume was extended to 10 mL with PBS 1X prior to a final centrifugation at 3000 *g* for 5 min, 4 °C. Then, in each well of a 96-well plate, 90 µL of the collected supernatants was dispensed and 10 µL of test compounds/ingredients at various concentrations. The plate was then incubated for 10 min prior to the addition of 100 µL of L-DOPA at 4 mM in PBS 1X. The formation of black/brown products was determined by measuring the absorbance in kinetic mode (37 °C, 1 measure each 30 min for 18 h) at 600 nm using a Multiskan SkyHigh microplate reader (Thermo Scientific, Waltham, MA, USA). The slope of appearance for black/brown products was calculated after 4 h of incubation at 37 °C. Each experiment was performed three times (N = 3). The obtained data were normalized and presented as curves defined between 0 and 100% activity, related to appropriated blank and control experiments. Kojic acid was used as reference for tyrosinase inhibition (Sigma-Aldrich, K3125).

Cell cytotoxicity. HaCaT cells were seeded in a 96 well plate at 5000 cells/well in a final volume of 200 μL of complete medium. After 48 h of incubation, cell culture medium was removed, and the extract to be tested was added at various concentration, diluted in DMEM without FBS. The toxicity of these compounds was evaluated after 24 h and 48 h of incubation by the AlamarBlue^TM^ Cell Viability Reagent (Invitrogen, Carlsbad, CA, USA). Briefly, compounds were removed, and cells were washed once with DPBS (with Ca^2+^/Mg^2+^) before the incubation at 37 °C with the AlamarBlue^TM^ reagent diluted to 1:10 in DPBS, final volume of 100 μL/well. After 2–3 h at 37 °C, the fluorescence of the 96-well plates were read with a Tecan INFINITE M PLEX 200 Pro plate reader (560/9 nm excitation wavelength, 590/20 nm emission wavelength). Cell viability was expressed in %, considering the untreated cells as the reference for 100% viability. All values were expressed as means ± SD.

Inflammatory conditioned-supernatant preparation. An amount of 150,000 HaCaT cells was seeded per well of a 6-well plate in 3 mL of complete cell culture medium. After 48 h of incubation at 37 °C, cell culture medium was changed for 1 mL of fresh serum-free medium containing the ingredients to test at 6 different concentrations starting from the dose limit of use (DLU) and diluted (DLU/2, DLU/4, DLU/8, DLU/16, DLU/32, corresponding, respectively, to 2.5, 1.25, 0.625, 0.313, 0.156, 0.078% *v*/*v*), with and without the combination with an inflammatory stimulation achieved by the addition of IFNγ (BioTechne, Noyal-Châtillon-sur-Seiche, France, 285-IF) and TNFα (BioTechne, 210-TA-020), 10 ng/mL final for each. After 48 h of additional incubation, all supernatants were collected and stored at −80 °C waiting for ELISA quantification of pro-inflammatory cytokines/chemokines. After rinsing with two washes of PBS, 3 mL/well, plates were kept at −80 °C while waiting for cell protein quantification by bicinchoninic acid assay (BCA assay).

ELISA quantification. ELISA quantifications were performed with Duoset kit from R&D systems (Minneapolis, MN, USA) regarding the secretion by cells of 9 cytokines and chemokines, all involved in inflammatory processes: CXCL9/MIG (ref. DY392), CCL5/RANTES (ref. DY278), IL-8/CXCL8 (ref. DY208), IL-33 (ref. DY3625B), IL-1 β/IL-1F2 (ref. DY201), IL-18 (ref. DY318), CCL17/TARC (ref. DY364), CXCL10/IP10 (ref. DY266), and IL-6 (ref. DY206). All ELISA were conducted according to the data sheet. Absorbances were read at 450 nm using a Multiskan SkyHigh microplate reader (Thermo Scientific). Results were expressed in pg of analytes, cytokine or chemokine, per mL of cell culture supernatant. All values were expressed as means ± SD.

Protein quantification. After collecting cell culture supernatants, the 6-well plates were washed once with DPBS (with Ca^2+^/Mg^2+^) before being frozen at −80 °C. Couple of freeze/thawing cycles were performed to lyse HaCaT cells. Then, cell proteins were quantified by BCA method according to the microplate procedure. After 30 min at 37 °C, the absorbance of the 96-well plates were read at 562 nm using a Multiskan SkyHigh microplate reader (Thermo Scientific). Protein concentrations were determined using a BSA-based calibration curve and were expressed in µg/mL. All values were expressed as means ± SD.

ELISA normalization. ELISA values expressed in pg of analytes per mL of cell culture supernatants were reported to BCA values expressed in µg of cell proteins per mL of cell lysates. Normalized data are expressed in pg of analytes per µg of cell proteins.

$$\frac{pg\;of\;analytes}{µg\;of\;cell\;proteins}=\frac{ELISA\;value\left(pg/mL\right)\times cell\;culture\;supernatent\left(mL\right)}{BCA\;value\left(µg/mL\right)\times cell\;lysate\;volume\;\left(mL\right)}$$

Error propagation for normalized ELISA. To display error bars for normalized ELISA data (z), a propagation of errors was calculated from standard deviations and means of ELISA values (x) and proteins quantification values (y). By considering the ELISA value (x) and its standard deviation (Δx), the associated proteins quantification value (y) and its standard deviation (Δy), the normalized ELISA value (z), and its relative error (Δz) to be determined, the following relation can be written:

$$\frac{\Delta z}{z}=\sqrt{{\left(\frac{\Delta x}{x}\right)}^{2}+{\left(\frac{\Delta y}{y}\right)}^{2}}$$

Therefore,

$$\Delta z=z.\sqrt{{\left(\frac{\Delta x}{x}\right)}^{2}+{\left(\frac{\Delta y}{y}\right)}^{2}}$$

Relative Quantification of MT1 Receptor by Flow Cytometry. HaCaT cells were incubated for 48 h with *C. islandica* extract without reaching the confluency. *C. islandica* extract was evaluated at its DLU, 2.5% (*v*/*v*), and half of it, 1.25% (*v*/*v*), mentioned as DLU/2. After the incubation time, cells were washed two times with Dulbecco’s phosphate buffered saline (DBPS) without Ca^2+^/Mg^2+^ prior being detached with 3 mL of Accutase (Sigma-Aldrich, #SCR005), 10 min at 37 °C. Once detached and washed with 2 mL of FACS buffer (Fluorescence-Activated Cell Sorting buffer, which is composed of DPBS without Ca^2+^/Mg^2+^ supplemented with FBS 10% (*v*/*v*)), 1 × 10^6^ HaCaT cells were incubated for 30 min at 4 °C with 300 µL of FACS buffer. Then, the mouse monoclonal IgG2b primary antibody against human MT1-MMP (BioTechne, MAB9181-100) was added at a final concentration of 0.8 µg/mL in 300 µL of FACS buffer for 1 h of incubation at 4 °C. An isotype control antibody (BioTechne, MAB0041) was realized at the same concentration. After two washes with 2 mL of FACS buffer, 10 µL of goat APC-conjugated anti-mouse IgG secondary antibody (BioTechne, F0101B) were added in a final volume of 300 µL. A control secondary antibody was realized with cells incubated only with the APC-conjugated anti-mouse IgG secondary antibody at the same concentration. After two final washing steps with 2 mL each, propidium iodide (PI, Sigma-Aldrich, 537060) was added to cell suspension at a final concentration of 1 µg/mL just before recording. White (unlabeled) cells and APC-labeled cells were analyzed using a BD LSR Fortessa X20 flow cytometer (Becton Dickinson, Franklin Lakes, NJ, USA), and 10,000 events were collected for processing with DiVa software (version 8.0.1). The isotype control cells were set at 2 × 10^2^ as mean fluorescence intensity for each condition of incubation, either not treated or treated with *C. islandica* extract. Only alive cells were recorded by gating on only PI-negative cells. Results are presented as the difference in fluorescence relative intensity (geometric mean) between the cells labeled with the anti-MT1 antibody versus the cells labeled with the corresponding isotype control antibody, expressed in expression fold change regarding untreated cells ± SD.

Statistical analysis. All values were expressed as means ± SD. Statistical analyses were conducted by one-way ANOVA tests (GraphPad Prism software, version 11.0.2 (92)) followed by a Dunnett’s multiple-comparison post hoc test. Group differences resulting in *p*-value < 0.05 by the one-way ANOVA were considered statistically significant and appear with * *p* < 0.05, ** *p* < 0.01, *** *p* < 0.001, and **** *p* < 0.0001. “ns” for no statistical significance correspond to *p* > 0.05.

## Data Availability

Data is contained within the article or Supplementary Materials.

## Supplementary Materials

The following supporting information can be downloaded at https://www.mdpi.com/article/10.3390/molecules31172988/s1, Figure S1: CCL5 production by HaCaT cells, challenged or not with TNFα + IFNγ (10 ng/mL), and incubated with various concentration of *C. islandica* hydroglycolic extract during 48 h. Production was measured by ELISA and normalized by cellular proteins amount. Data are expressed in ng of cytokines/chemokines per mg of cell proteins. Values are means ± SD (*n* = 3); Figure S2: CXCL9 production by HaCaT cells, challenged or not with TNFα + IFNγ (10 ng/mL), and incubated with various concentration of *C. islandica* hydroglycolic extract during 48 h. Production was measured by ELISA and normalized by cellular proteins amount. Data are expressed in ng of cytokines/chemokines per mg of cell proteins. Values are means ± SD (*n* = 3); Figure S3: CXCL10 production by HaCaT cells, challenged or not with TNFα + IFNγ (10 ng/mL), and incubated with various concentration of *C. islandica* hydroglycolic extract during 48 h. Production was measured by ELISA and normalized by cellular proteins amount. Data are expressed in ng of cytokines/chemokines per mg of cell proteins. Values are means ± SD (*n* = 3); Figure S4: IL6 production by HaCaT cells, challenged or not with TNFα + IFNγ (10 ng/mL), and incubated with various concentration of *C. islandica* hydroglycolic extract during 48 h. Production was measured by ELISA and normalized by cellular proteins amount. Data are expressed in ng of cytokines/chemokines per mg of cell proteins. Values are means ± SD (*n* = 3); Figure S5: IL8 production by HaCaT cells, challenged or not with TNFα + IFNγ (10 ng/mL), and incubated with various concentration of *C. islandica* hydroglycolic extract during 48 h. Production was measured by ELISA and normalized by cellular proteins amount. Data are expressed in ng of cytokines/chemokines per mg of cell proteins. Values are means ± SD (*n* = 3); Figure S6: Modulation of MT1 protein expression by HaCaT cells assessed by flow cytometry after 48 h of incubation with *C. islandica* hydroglycolic extract. Results are expressed as expression fold change relative to not treated cells. The extract was investigated at the DLU and DLU/2 corresponding respectively to 2.5% and 1.25% (*v/v*). (N = 5, *n* = 10,000); Figure S7: HPLC Characterization of *C. Islandica* hydroglycolic extract performed by LCTECH company. HPLC fingerprints were created using regular HPLC protocol, which provides a straightforward method for identification and characterization. Slight changes may occur due to the natural origin of the product. More details are provided in Figure S8; Figure S8: Experiment details from LCTECH to obtain the chromatogram presented in Figure S7.

## Abbreviations

The following abbreviations are used in this manuscript:

INRAE	Institut National de Recherche pour l’Agriculture, l’Alimentation et l’Environnement.
UMR	Unité Mixte de Recherche
IL-6	Interleukine-6
IL-8	Interleukine-8 (CXCL8)
MIG	Monokine Induced by Gamma (CXCL9)
IP-10	Interferon γ-induced Protein 10 kDa (CXCL10)
RANTES	Regulated on Activation, Normal T-cell Expressed and Secreted (CCL5)
MT1-MMP	Membrane Type 1 Metalloproteinase (MT1-MMP/MMP14)
CuPRAC	CUPric Reducing Antioxidant Capacity
TEAC	Trolox Equivalent Antioxidant Capacity
UV	Ultraviolet
DLU	Dose Limit of Use
IFNγ	Interferon γ
TNFα	Tumor Necrosis Factor α
ELISA	Enzyme-Linked Immunosorbent Assay
CD44	Cluster of Differentiation 44
FACS	Fluorescence-Activated Cell Sorting
IC50	Inhibitory Concentration 50
ATCC	American Type Culture Collection
DMEM	Dulbecco’s Modified Eagle Medium
FBS	Fetal Bovine Serum
PBS	Phosphate Buffer Saline
SD	Standard Deviation
BCA	Bicinchoninic Acid
DPBS	Dulbecco’s Phosphate-Buffered Saline
BSA	Bovine Serum Albumin
Ig	Immunoglobulin
APC	Allophycocyanin
PI	Propidium Iodide
PIF	Product Information File
HPLC	High-Performance Liquid Chromatography
