# Supplementary material for: In Vitro Anti-Aging Properties of a Cetraria islandica Extract: Interest for Skin Care Cosmetics
Source: Molecules. 2026 Aug 26;31(17):2988. doi: 10.3390/molecules31172988 (PMC13567190; doi:10.3390/molecules31172988)
Supplement: Supplementary file 1 [file molecules-31-02988-s001.zip › molecules-4397537-supplementary.pdf]

molecules

Supplementary Information

In Vitro Anti-Aging Properties of a *Cetraria islandica* Extract: Interest for Skin Care Cosmetics

Anthony Groso [1], Paul Jabet [1], Yang Fan [2,*], Wang Hua [2], Hu Yawen [2], Guo Miao [2], Richard Daniellou [1,3,*] and Guillaume Collet [1,*]

1 Chair of Cosmetology, AgroParisTech, 45100 Orléans, France
2 Research & Development Center, Mageline Biology Tech Co., Ltd., Wuhan, Hubei, China
3 INRAE, AgroParisTech, UMR Micalis, Université Paris-Saclay, 78350 Jouy-en-Josas, France
* Correspondence: yangfan@mageline.cn (Y.F.); richard.daniellou@agroparistech.fr (R.D.); guillaume.collet@agroparistech.fr (G.C.)

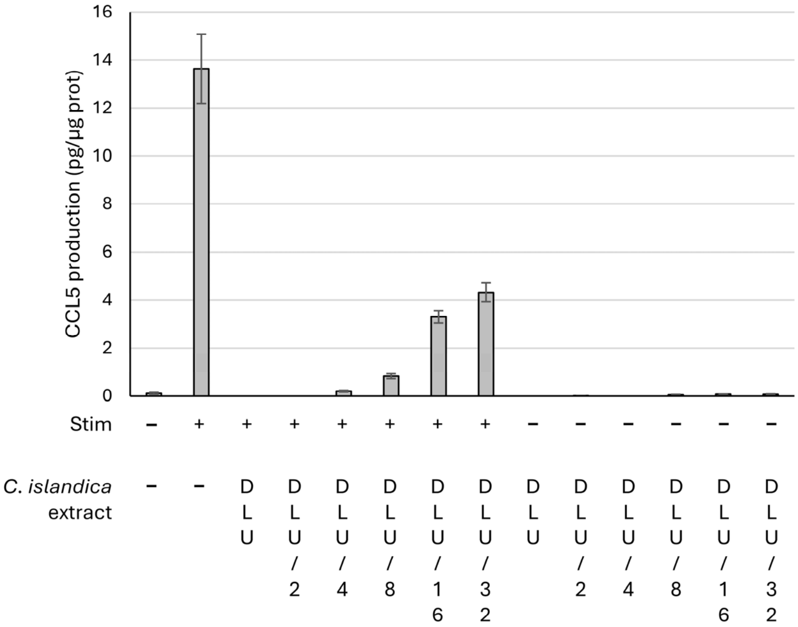

Figure S1. CCL5 production by HaCaT cells, challenged or not with TNFα + IFNγ (10 ng/mL), and incubated with various concentration of *C. islandica* hydroglycolic extract during 48 h. Production was measured by ELISA and normalized by cellular proteins amount. Data are expressed in ng of cytokines/chemokines per mg of cell proteins. Values are means ± SD (*n* = 3).

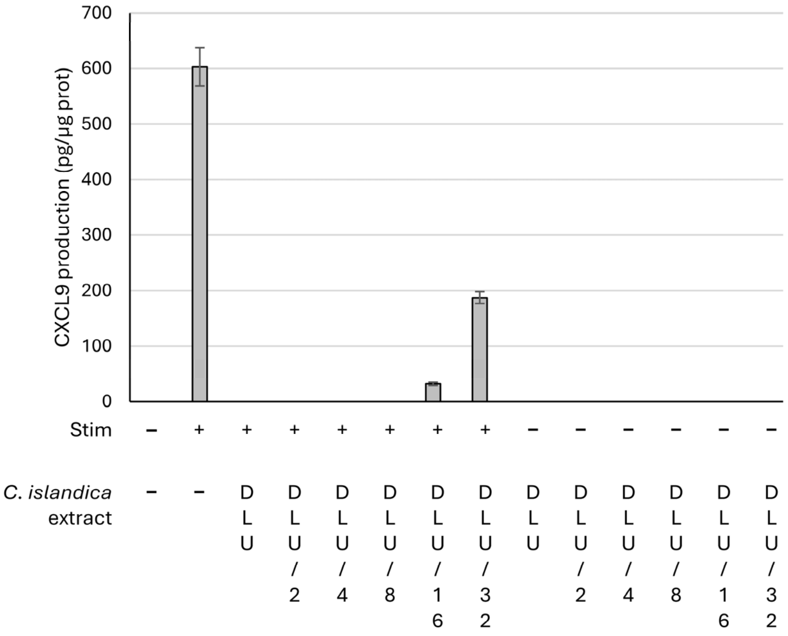

Figure S2. CXCL9 production by HaCaT cells, challenged or not with TNFα + IFNγ (10 ng/mL), and incubated with various concentration of *C. islandica* hydroglycolic extract during 48 h. Production was measured by ELISA and normalized by cellular proteins amount. Data are expressed in ng of cytokines/chemokines per mg of cell proteins. Values are means ± SD (n = 3).

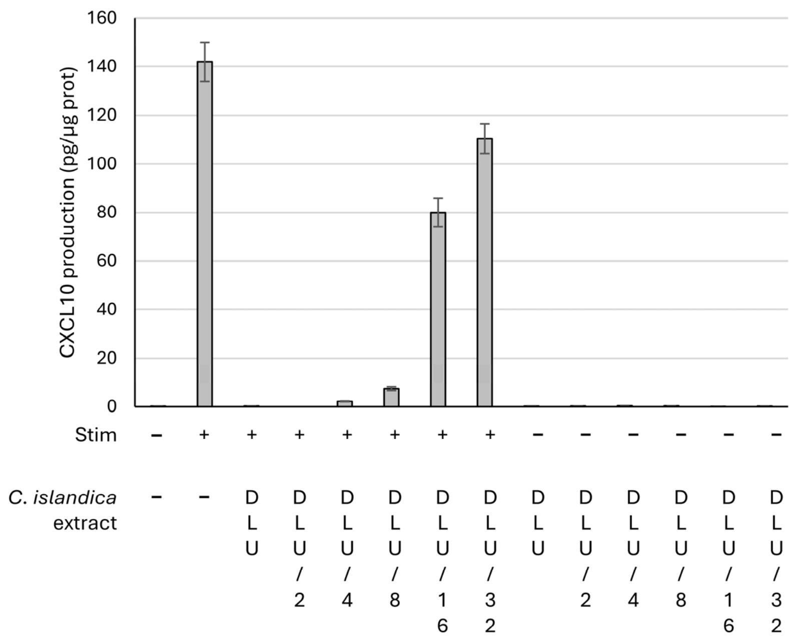

Figure S3. CXCL10 production by HaCaT cells, challenged or not with TNFα + IFNγ (10 ng/mL), and incubated with various concentration of *C. islandica* hydroglycolic extract during 48 h. Production was measured by ELISA and normalized by cellular proteins amount. Data are expressed in ng of cytokines/chemokines per mg of cell proteins. Values are means ± SD (*n* = 3).

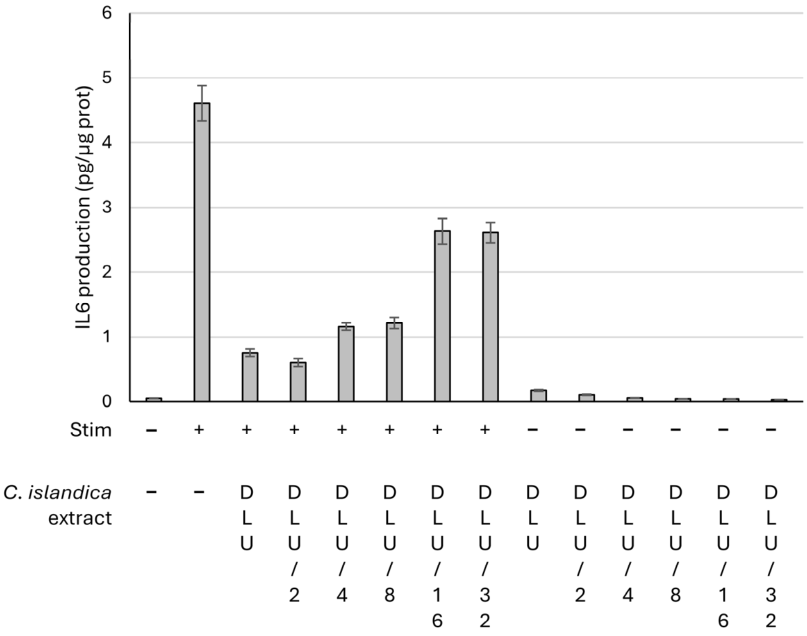

Figure S4. IL6 production by HaCaT cells, challenged or not with TNFα + IFNγ (10 ng/mL), and incubated with various concentration of *C. islandica* hydroglycolic extract during 48 h. Production was measured by ELISA and normalized by cellular proteins amount. Data are expressed in ng of cytokines/chemokines per mg of cell proteins. Values are means ± SD (*n* = 3).

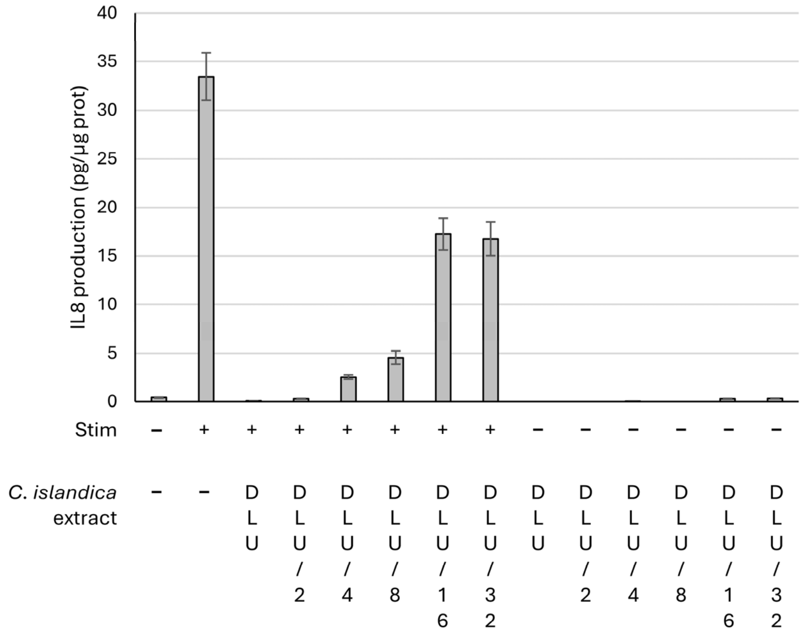

Figure S5. IL8 production by HaCaT cells, challenged or not with TNFα + IFNγ (10 ng/mL), and incubated with various concentration of *C. islandica* hydroglycolic extract during 48 h. Production was measured by ELISA and normalized by cellular proteins amount. Data are expressed in ng of cytokines/chemokines per mg of cell proteins. Values are means ± SD (*n* = 3).

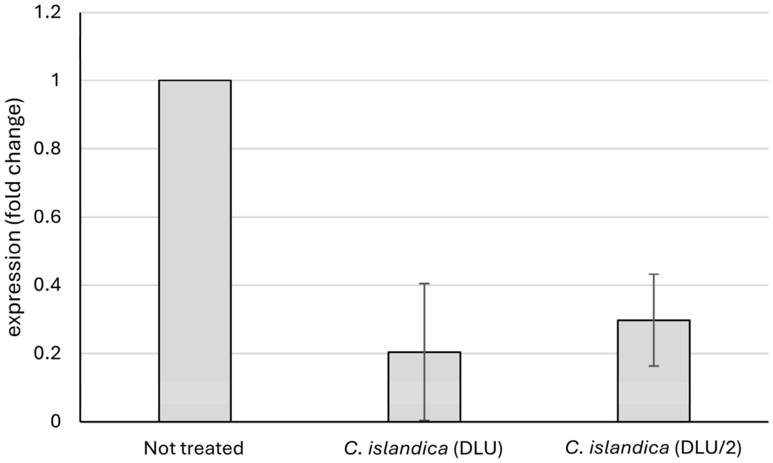

Figure S6. Modulation of MT1 protein expression by HaCaT cells assessed by flow cytometry after 48 h of incubation with *C. islandica* hydroglycolic extract. Results are expressed as expression fold change relative to not treated cells. The extract was investigated at the DLU and DLU/2 corresponding respectively to 2.5 % and 1.25 % (*v*/*v*). (N = 5, *n* = 10,000).

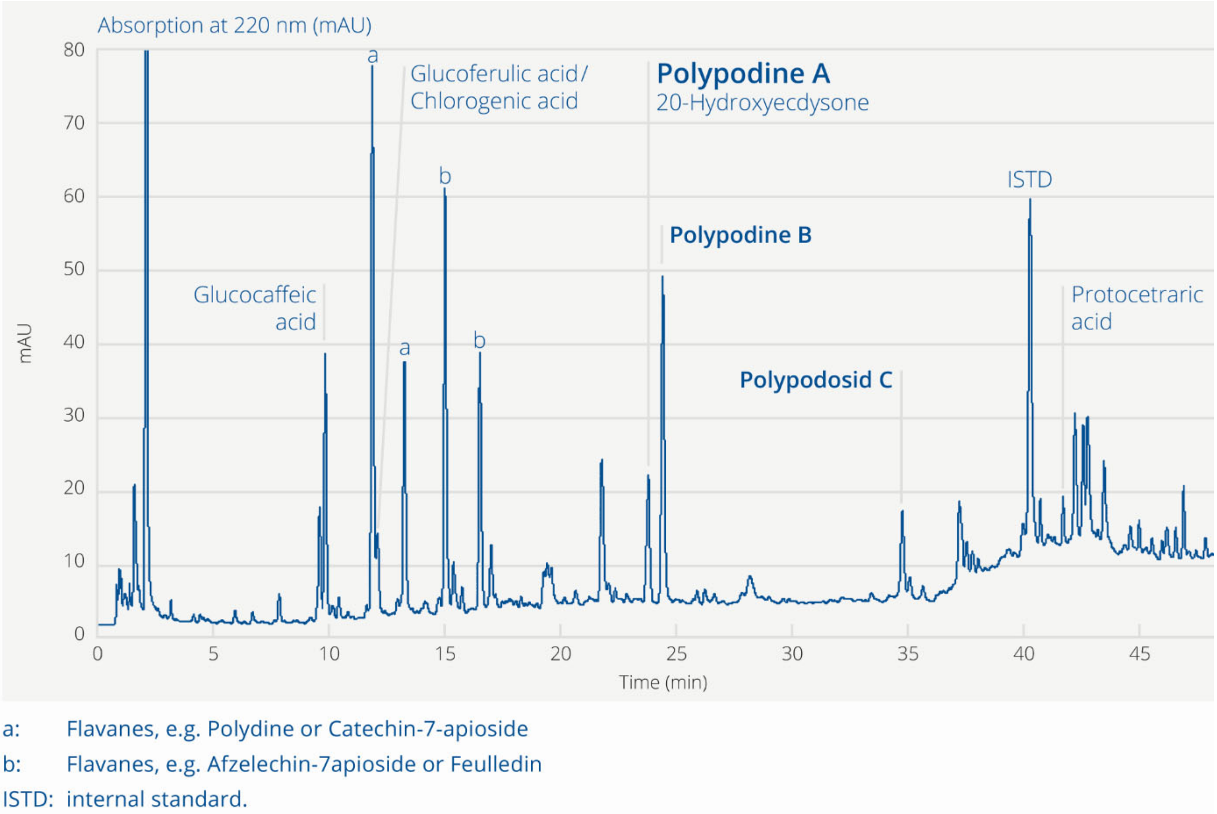

Figure S7. HPLC Characterization of *C. Islandica* hydroglycolic extract performed by LCTECH company. HPLC fingerprints were created using regular HPLC protocol, which provides a straightforward method for identification and characterization. Slight changes may occur due to the natural origin of the product. More details are provided in Figure S8.

Method origin. The HPLC-DAD method was developed in-house by LCTECH based on conventional reversed-phase chromatographic principles for the qualitative fingerprinting of lichen-derived secondary metabolites. The procedure was intended for product characterization and batch-to-batch comparison rather than for the quantitative determination of individual constituents.

Sample preparation. An accurately weighed portion of the *Cetraria islandica* extract (0.20 g) was mixed with 10 mL of acetone and sonicated for 40 min at 30 °C. After cooling to room temperature, any solvent loss was compensated with acetone. The mixture was allowed to settle, and the supernatant was passed through a 0.45 μm PTFE membrane filter before HPLC injection.

Chromatographic conditions. Chromatographic separation was performed on a reversed-phase C18 column (250 × 4.6 mm, 5 μm) maintained at 30 °C. The mobile phase consisted of methanol (eluent A) and 0.1% (v/v) formic acid in water (eluent B). The flow rate was 1.0 mL/min, and the injection volume was 20 μL. Gradient elution was applied as shown below.

Time (min)	Eluent A (%)	Eluent B (%)	Comment
0	40	60	
15	65	35	
35	85	15	
45	95	5	
50	95	5	End of analytical acquisition
52	40	60	Return to initial conditions
60	40	60	8 min re-equilibration

DAD detection. The representative qualitative fingerprint chromatogram was recorded at 220 nm, consistent with the chromatogram presented in the analytical report. Additional DAD channels at 245, 254 and 275 nm were used as supplementary wavelengths for the observation of selected lichen-derived constituents. The analytical acquisition time was 50 min, followed by return to the initial mobile-phase composition and an 8 min column re-equilibration period.

Reference standards and other relevant substances. Orcinol and (+)-usnic acid were used as reference standards and were dissolved in acetone to prepare a mixed reference solution. HPLC-grade methanol, formic acid and acetone, together with ultrapure water, were used for sample preparation and chromatographic analysis.

Figure S8. Experiment details from LCTECH to obtain the chromatogram presented in Figure S7.
